# Dental pulp-derived stem cell conditioned medium reduces cardiac injury following ischemia-reperfusion

**DOI:** 10.1038/srep16295

**Published:** 2015-11-06

**Authors:** Satoshi Yamaguchi, Rei Shibata, Noriyuki Yamamoto, Masaya Nishikawa, Hideharu Hibi, Tohru Tanigawa, Minoru Ueda, Toyoaki Murohara, Akihito Yamamoto

**Affiliations:** 1 Department of Oral and Maxillofacial Surgery, Nagoya University Graduate School of Medicine, Nagoya, Japan; 2 Department of Cardiology, Nagoya University Graduate School of Medicine, Nagoya, Japan; 3 Department of Otolaryngology, Aichi Medical University, Nagakute, Japan

## Abstract

Stem cells from human exfoliated deciduous teeth (SHEDs) can regenerate various tissues. We investigated the impact of SHED-conditioned medium (SHED-CM) on myocardial injury in a mouse model of ischemia-reperfusion (I/R). Wild-type (WT) mice were subjected to myocardial ischemia followed by reperfusion. SHED-CM was intravenously injected at 5 min after reperfusion. Administration of SHED-CM reduced myocardial infarct size as well as decreased apoptosis and inflammatory cytokine levels, such as TNF-α, IL-6, and IL-β, in the myocardium following I/R. In cultured cardiac myocytes, SHED-CM significantly suppressed apoptosis under hypoxia/serum-deprivation and reduced LPS-induced expression of pro-inflammatory genes. Furthermore, anti-apoptotic action of SHED-CM was stronger than bone marrow-derived stem cell (BMSC)-CM or adipose-derived stem cell (ADSC)-CM in cardiac myocytes. SHED-CM contains a higher concentration of hepatocyte growth factor (HGF) than BMSC-CM and ADSC-CM, and neutralization of HGF attenuated the inhibitory actions of SHED-CM on apoptosis in cardiac myocytes. Finally, WT mice were intravenously treated with an HGF-depleted SHED-CM, followed by myocardial I/R. HGF depletion significantly attenuated the inhibitory actions of SHED-CM on myocardial infarct size and apoptosis after I/R. SHED-CM protects the heart from acute ischemic injury because it suppresses inflammation and apoptosis. SHED-CM could be a useful treatment option for acute myocardial infarction.

Ischemic heart diseases, such as myocardial infarction (MI), are some of the leading causes of death worldwide^1^. Even after MI treatment with current percutaneous coronary intervention and pharmacological therapies, which have been shown to limit infarct size and preserve cardiac function, MI usually causes irreversible damage to heart tissues, such that mortality and morbidity remain high^2^. Therefore, the development of effective adjunctive therapies for patients with MI is still necessary.

In recent years, stem cell therapy has been investigated as a new strategy for regenerative medicine^3^. Dental pulp-derived stem cells such as human adult dental pulp stem cells (DPSCs) and stem cells from human exfoliated deciduous teeth (SHEDs) are self-renewing mesenchymal stem cells (MSCs) residing within the perivascular niche of the dental pulp^4^,^5^. They are thought to originate from the cranial neural crest, which expresses early markers for both MSCs and neuroectodermal stem cells^4^,^5^,^6^. DPSCs and SHEDs have been reported to demonstrate the ability to regenerate various tissues. We have recently shown that implantation of DPSCs or SHEDs promotes functional recovery after spinal cord injury^6^,^7^. DPSCs also protect against ischemic brain injury in neonatal mice^8^,^9^. Recent studies have reported improved cardiac function after the administration of DPSCs to treat MI in rat model^10^. Thus, cell therapy using dental pulp-derived stem cells is a promising treatment for regenerative medicine. However, cell transplantation has several disadvantages. For clinical use, cells must be expanded by a reliable cell culture system that produces sufficient cell numbers for clinical effects while also meeting safety requirements, and tumorigenesis and strong immune reactions must be avoided^11^,^12^.

One of the major mechanisms underlying the beneficial effect of cell therapy is the release of multiple cytokines such as VEGF. Recently, it has been reported that conditioned media derived from various stem cells prevents acute organ damage^13^,^14^,^15^,^16^. We have also shown that SHED transplantation and administration of serum-free cultured conditioned medium (CM) from SHEDs (SHED-CM) demonstrate similar endogenous tissue regenerative activity in the treatment of spinal cord injury^7^,^17^. Theoretically, the use of allogeneic CM can circumvent ethical controversies and immune-related problems. Administration of preserved CM is convenient for immediate application and minimizes surgical invasiveness. Here, we investigated the effect of SHED-CM on acute ischemic injury in the heart *in vivo*. We also tested whether SHED-CM modulates cardiac myocytes behavior *in vitro* and evaluated its possible mechanisms.

## Results

### Systemic delivery of SHED-CM reduces myocardial infarct size in mice following ischemia/reperfusion

**Figure 1:**
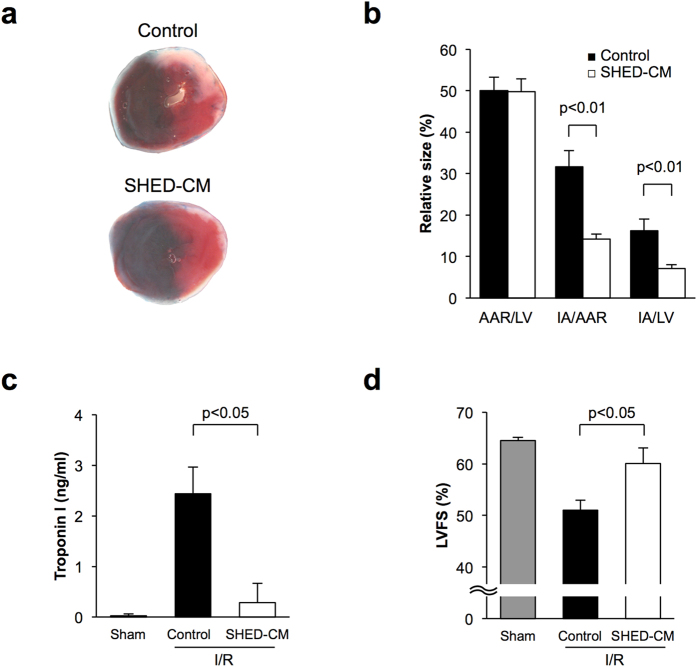
SHED-CM reduces myocardial infarct size in mice following ischemia-reperfusion. (**a**) Representative pictures of myocardial tissues from mice treated with control or SHED-CM at 24 h after ischemia-reperfusion. The nonischemic area is indicated by blue, the area at risk (AAR) by red, and the infarct area (IA) by white. (**b**) Quantification of LV area, AAR, and IA in WT mice treated with control (n = 6) and SHED-CM (n = 6). (**c**) Plasma levels of cardiac troponin I, a highly specific marker for cardiac injury, of mice treated with control (n = 5) and SHED-CM (n = 5) at 24 h after sham operation or myocardial ischemia-reperfusion. Results are presented as mean ± SEM. (**d**) Left ventricular fractional shortening assessed by echocardiography treated with control (n = 4) or SHED-CM (n = 4), at 7 days after myocardial ischemia-reperfusion.

To test whether systemic delivery of SHED-CM affects acute cardiac ischemic injury in mice, we subjected wild-type (WT) C57BL/6J mice to 30 minutes of myocardial ischemia and 24 h of reperfusion. Five minutes after reperfusion, mice were intravenously treated with SHED-CM or DMEM as a control. All mice survived after the surgical induction of ischemia-reperfusion (I/R), and body weight and blood pressure did not differ between the two experimental groups (data not shown). Figure 1a shows representative photographs of heart tissues stained with Evans blue dye to delineate the area at risk (AAR) and 2,3,5-triphenyl tetrazolium chloride (TTC) to delineate the infarct area (IA). Systemic delivery of SHED-CM significantly attenuated the IA/AAR and IA/LV ratios by 55.1% and 55.9%, respectively, compared with control (Fig. 1b). In contrast, AAR/LV did not differ between the two experimental groups. The circulating level of troponin I, a marker of heart injury, was significantly lower in SHED-CM-treated mice than in control mice at 24 h after reperfusion (Fig. 1c). Furthermore, treatment with SHED-CM significantly increased left ventricular fractional shortening in mice at 7 days after myocardial I/R as measured by echocardiography (Fig. 1d).

### Delivery of SHED-CM suppresses apoptosis and inflammatory responses in ischemic heart *in vivo*

**Figure 2:**
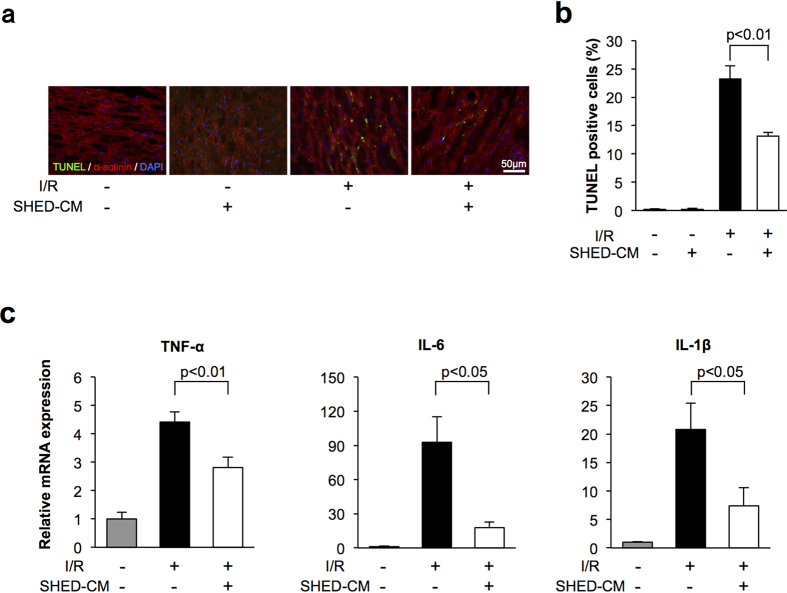
SHED-CM suppressed apoptosis and inflammation in mice following myocardial ischemia-reperfusion. (**a**) Representative photographs of heart sections stained with TUNEL from WT mice treated with control and SHED-CM at 24 h after sham operation or myocardial ischemia-reperfusion. Apoptotic nuclei were determined by TUNEL staining (green), and cardiac myocytes were stained with sarcomeric α-actinin (red). Total nuclei were counterstained with DAPI (blue). (**b**) Quantitative analysis of apoptotic nuclei from WT mice treated with control (n = 5) and SHED-CM (n = 5) at 24 h after sham operation or myocardial ischemia-reperfusion. TUNEL-positive cells were counted in three randomly chosen microscopic fields from five different sections in each tissue block and expressed as a percentage of the total number of nuclei. (**c**) mRNA levels of TNF-α, IL-6, and IL-1β in the myocardium in WT mice treated with control and SHED-CM at 24 h after myocardial ischemia-reperfusion. mRNA levels were measured by real-time PCR (n = 5 in each group). All results are normalized to GAPDH. Results are presented as mean ± SEM.

Apoptosis is the key feature of ischemic heart disease^18^. To investigate the impact of SHED-CM on myocyte apoptosis in the heart, we stained heart sections with terminal deoxynucleotidyl transferase-mediated dUTP nick-end labeling (TUNEL) and sarcomeric actinin at 24 h after I/R or sham operation. Figure 2a shows representative fluorescent photographs of TUNEL-positive nuclei in the AAR. Quantitative analysis indicated that SHED-CM significantly reduced the frequency of TUNEL-positive myocytes in the ischemic heart compared to that with control treatment (Fig. 2b). Few to no TUNEL-positive myocytes were observed in the heart of control or SHED-CM-treated mice after sham operation.

Because inflammation contributes to myocardial injury after I/R^19^, the expression of proinflammatory cytokines including tumor necrosis factor-alpha (TNF-α), interleukin-6 (IL-6), and interleukin 1-beta (IL-1β) was evaluated in ischemic hearts from control or SHED-CM-treated mice by quantitative RT-PCR. Cardiac TNF-α, IL-6, and IL-1β mRNA levels in WT mice were elevated by I/R injury, but these inductions were attenuated by SHED-CM administration (Fig. 2c and Supplemental Figure 1).

### SHED-CM attenuates apoptosis and inflammatory responses in cardiac myocytes *in vitro*

**Figure 3:**
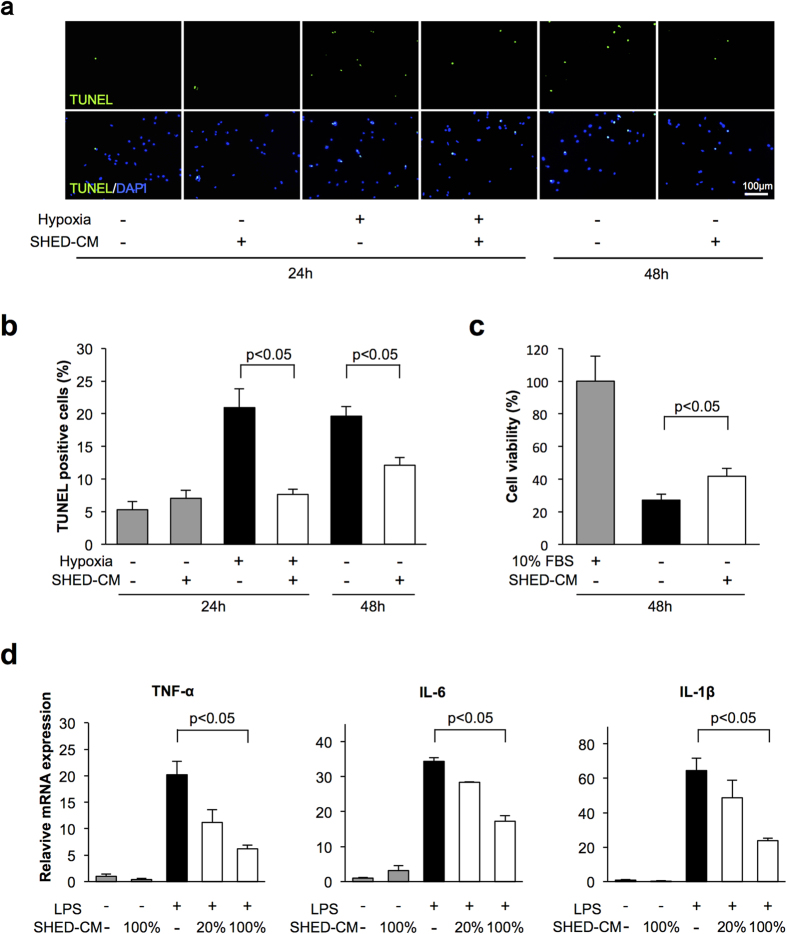
SHED-CM attenuates apoptosis and inflammation in cardiac myocytes. Rat neonatal cardiac myocytes were treated with control and SHED-CM under 24 h of hypoxia or 48 h of normoxic conditions. (**a**) Representative photomicrographs of TUNEL-positive cardiac myocytes. Apoptotic nuclei were identified by TUNEL-staining (green) and total nuclei by DAPI counterstaining (blue). (**b**) Quantitative analysis of TUNEL-positive cardiac myocytes treated with vehicle and SHED-CM under 24 h of hypoxia or 48 h of normoxic conditions. TUNEL-positive cells were counted in three randomly chosen microscopic fields of the three different slides and expressed as a percentage of the total number of nuclei. (**c**) Cell viability of cardiac myocytes was analyzed by WST-8 assay after 48 h serum deprivation treated with vehicle and SHED-CM. Results are presented as mean ± SEM (n = 3 in each group). (**d**) Effect of SHED-CM on LPS-induced expression of TNF-α, IL-6, and IL-1β in cardiac myocytes. Cardiac myocytes were pretreated with vehicle, 20% SHED-CM or SHED-CM for 1 h and stimulated with or without LPS (100 ng/ml) for 6 h. The mRNA expression of cytokines was measured by real-time PCR and expressed relative to GAPDH levels (n = 3 in each group). Results are presented as mean ± SEM.

To examine the effect of SHED-CM on apoptosis at a cellular level, neonatal rat cardiac myocytes were subjected to normoxia or hypoxia under conditions of serum-deprivation in the presence of SHED-CM or vehicle. Treatment with SHED-CM diminished the frequency of TUNEL-positive cells under condition of serum-deprivation for 48 h by 38.2%. Hypoxia for 24 h increased the frequency of TUNEL-positive cardiac myocytes, and SHED-CM suppressed the frequency of TUNEL-positive cardiac myocytes under conditions of hypoxia by 63.5% (Fig. 3a,b). Furthermore, we assessed cell viability after 48 h serum-deprivation by WST-8 assay. Serum-deprivation led to an increase in the ratio of dead cells among cardiac myocytes. SHED-CM treatment improved cell viability under serum-deprived conditions (Fig. 3c).

To analyze the anti-inflammatory actions of SHED-CM at a cellular level, cardiac myocytes were pretreated with SHED-CM or vehicle followed by stimulation with lipopolysaccharide (LPS). LPS exposure increased mRNA levels of TNF-α, IL-6, and IL-1β in cardiac myocytes. SHED-CM treatment dose-dependently suppressed the LPS-induced increases in TNF-α, IL-6, and IL-1β expression (Fig. 3d and Supplemental Figure 1).

### SHED-CM treatment attenuates apoptosis more effectively than BMSC-CM or ADSC-CM treatment in cardiac myocytes

**Figure 4:**
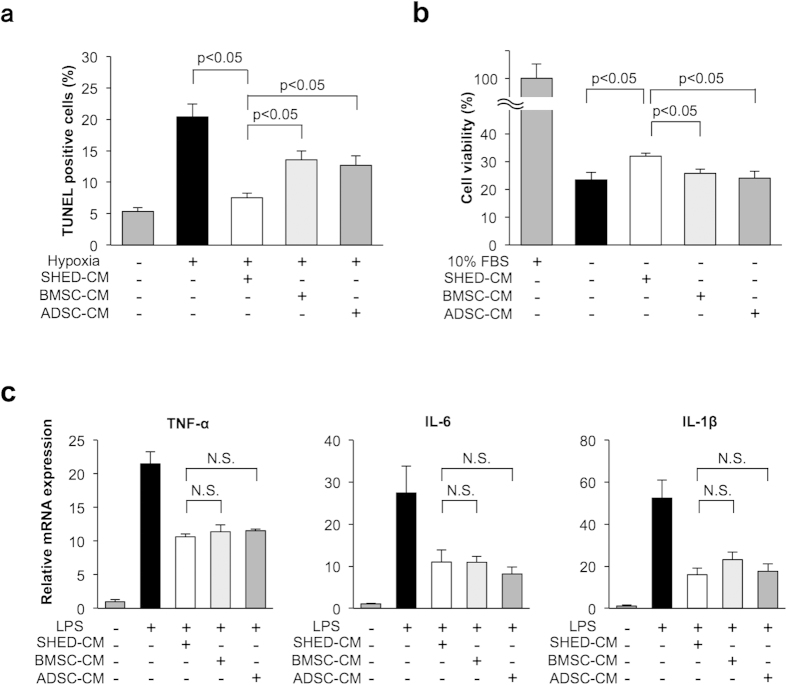
SHED-CM attenuates apoptosis more effectively than BMSC-CM or ADSC-CM in cardiac myocytes. Cardiac myocytes were treated with SHED-CM, BMSC-CM, or ADSC-CM under 24 h of hypoxia or 48 h of normoxia. (**a**) Quantitative analysis of TUNEL-positive cardiac myocytes treated with SHED-CM, BMSC-CM, or ADSC-CM under 24 h of hypoxia. TUNEL-positive nuclei were counted in three randomly selected microscopic fields of the three different slides and expressed as a percentage of the total number of nuclei (n = 3 in each group). (**b**) Cell viability of cardiac myocytes was analyzed by WST-8 assay after 48-h serum deprivation treated with SHED-CM, BMSC-CM, or ADSC-CM. Results are presented as mean ± SEM (n = 3 in each group). Control cells were cultured in DMEM containing 10% FBS. The numbers of surviving cardiac myocytes were expressed as percentage of control cells. (**c**) Effect of SHED-CM, BMSC-CM, or ADSC-CM on LPS-induced expression of TNF-α, IL-6, and IL-1β in cardiac myocytes. Cardiac myocytes were pretreated with SHED-CM, BMSC-CM, or ADSC-CM for 1 h and stimulated with LPS (100 ng/ml) for 6 h. The mRNA expression of cytokines was measured by real-time PCR and expressed relative to GAPDH levels (n = 3 in each group). Results are presented as mean ± SEM.

To compare the anti-apoptotic actions of CM derived from various mesenchymal stem cells, cardiac myocytes were subjected to hypoxia under conditions of serum deprivation in the presence of SHED-CM, human bone marrow-derived stem cell (BMSC)-CM, human adipose tissue-derived stem cell (ADSC)-CM, or vehicle. SHED-CM, BMSC-CM, and ADSC-CM diminished the frequency of TUNEL-positive cells under hypoxic conditions (Fig. 3a). Notably, treatment with SHED-CM was associated with a significant decrease in the proportion of TUNEL-positive apoptotic cells in cardiac myocytes compared to that with BMSC-CM or ADSC-CM treatment (Fig. 4a). In addition, SHED-CM improved cell viability under serum-deprived condition as measured by WST-8 assay (Fig. 4b). BMSC-CM and ADSC-CM did not affect cell viability under serum-deprived condition (Fig. 4b). The degree of inhibitory action on LPS-induced inflammatory cytokines was similar in the three groups (Fig. 4c and Supplemental Figure 1). Collectively, these results suggested that SHED-CM treatment attenuated apoptosis more effectively than BMSC-CM or ADSC-CM treatment in cardiac myocytes.

### Depletion of HGF attenuated the inhibitory actions of SHED-CM on apoptosis

**Figure 5:**
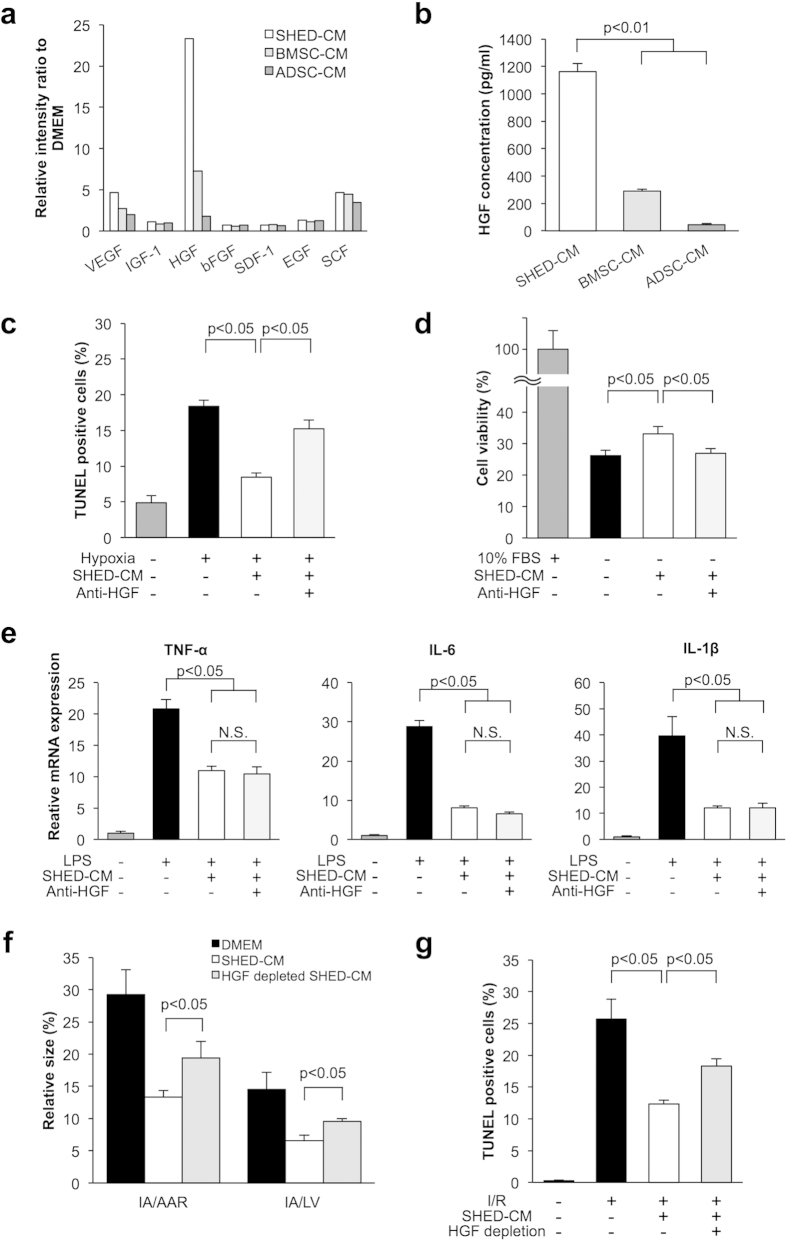
(**a**) Cytokine antibody array analysis of SHED-CM, BMSC-CM, and ADSC-CM. The relative intensity of multiple cytokines is shown. (**b**) Quantitative analysis of HGF concentrations in SHED-CM, BMSC-CM, and ADSC-CM by ELISA (n = 3 in each group). (**c**) Quantitative analysis of TUNEL-positive cardiac myocytes treated with SHED-CM or SHED-CM containing anti-human HGF neutralizing antibody (1 μg/ml) or control IgG under 24 h of hypoxia. TUNEL-positive nuclei were counted in three randomly selected microscopic fields of the three different slides and expressed as a percentage of the total number of nuclei (n = 3 in each group). (**d**) Cell viability of cardiac myocytes was analyzed by WST-8 assay after 48-h serum deprivation treated with SHED-CM or SHED-CM containing anti-human HGF neutralizing antibody (1 μg/ml) or control IgG. Control cells were cultured in DMEM containing 10% FBS. The numbers of surviving cardiac myocytes were expressed as percentage of control cells (n = 3 in each group). (**e**) Effect of SHED-CM containing anti-human HGF neutralizing antibody (1 μg/ml) or control IgG on LPS-induced expression of TNF-α, IL-6, and IL-1β in cardiac myocytes. Cardiac myocytes were treated with CMs for 1 h, and stimulated with LPS (100 ng/ml) for 6 h. The mRNA expression of cytokines was measured by real-time PCR method and expressed relative to GAPDH levels (n = 3 in each group). (**f**) Quantification of infarct size in WT mice treated with control, SHED-CM, or HGF-depleted SHED-CM. WT mice were intravenously treated with control, SHED-CM, and HGF-depleted SHED-CM. HGF was specifically immunodepleted from SHED-CM (n = 5 in each group). (**g**) Quantitative analysis of apoptotic nuclei from WT mice treated with control, SHED-CM, or HGF-depleted SHED-CM at 24 h after myocardial ischemia-reperfusion (n = 3 in each group). TUNEL-positive cells were counted in three randomly chosen microscopic fields from three different sections in each tissue block and expressed as the number of TUNEL-positive cells. Results are presented as mean ± SEM.

To examine the possible mechanism underlying the strong anti-apoptotic activity of SHED-CM, we applied a screening approach using human cytokine antibody arrays. SHED-CM, BMSC-CM, and ADSC-CM expressed multiple cytokines including vascular endothelial growth factor (VEGF), insulin-like growth factor I (IGF-1), hepatocyte growth factor (HGF), basic fibroblast growth factor (bFGF), stromal cell-derived factor 1 (SDF-1), epidermal growth factor (EGF), and stem cell factor (SCF). Notably, HGF was a significantly more highly expressed in SHED-CM compared to BMSC-CM and ADSC-CM (Fig. 5a). We validated the high level of HGF in the culture medium by ELISA. SHED-CM contained a higher concentration of HGF than BMSC-CM and ADSC-CM (Fig. 5b).

To determine whether HGF is involved in the strong anti-apoptotic activity of SHED-CM, cardiac myocytes were preincubated with a neutralizing antibody against HGF or control IgG followed by treatment with SHED-CM. Pretreatment with anti-HGF antibody reversed the inhibitory effects of SHED-CM on apoptotic response to hypoxia (Fig. 5c). SHED-CM-induced enhancement of cell viability was also inhibited by anti-HGF antibody (Fig. 5d). In contrast, pretreatment with anti-HGF antibody did not affect the suppressive actions of SHED-CM on LPS-stimulated mRNA expression of TNF-α, IL-6, and IL-1β in cardiac myocytes (Fig. 5e and Supplemental Figure 1).

To further examine whether HGF participates in SHED-CM-mediated protection against I/R damage *in vivo*, WT mice were intravenously treated with HGF-depleted SHED-CM or SHED-CM at 5 minutes after reperfusion. HGF was specifically immunodepleted from SHED-CM, and the loss of HGF from SHED-CM was confirmed by ELISA (Supplemental Fig. 2). HGF depletion significantly attenuated the inhibitory actions of SHED-CM on the myocardial infarct area after I/R (Fig. 5f). HGF depletion also reversed the SHED-CM-induced decrease in TUNEL-positive myocytes in ischemic heart (Fig. 5g). Thus, depletion of HGF attenuated the inhibitory actions of SHED-CM on apoptosis in response to ischemia.

## Discussion

The present study provides the first evidence that CM derived from dental pulp confers resistance to acute ischemic damage in the heart. Administration of SHED-CM attenuated MI and improved cardiac function in mice after I/R, which was associated with suppression of apoptosis and inflammation in the ischemic heart. Our *in vitro* experiments demonstrated that SHED-CM promoted the survival of cardiac myocytes in response to hypoxia and serum-deprivation, and that SHED-CM attenuated LPS-stimulated expression of pro-inflammatory mediators. Thus, SHED-CM administration can protect the heart from ischemic damage through at least two mechanisms involving reduction of cardiomyocyte death and suppression of inflammatory responses in myocardial cells. Because therapeutic approaches to minimize cell death and inflammation in the heart are believed to be logical strategies to treat acute cardiac injury, administration of SHED-CM may be a useful adjunctive therapy for acute MI.

The increased production of pro-inflammatory cytokines is an important component of post-ischemic myocardial injury^19^. SHED-CM attenuated myocardial infarction in mice after I/R, which was associated with the suppression of inflammation in the ischemic heart. Our *in vitro* experiments also showed that SHED-CM attenuated agonist-stimulated expression of pro-inflammatory mediators in cardiac myocytes. The degree of inhibitory action on inflammatory cytokines was similar across treatments with SHED-CM, BMSC-CM, and ADSC-CM. These data suggest that suppression of inflammatory cytokines in the heart is a common mechanism of cardio-protective actions in therapies using CMs-derived from various MSCs.

Myocyte apoptosis is a key feature in heart disorders involving ischemic heart disease^18^. The limitation of apoptosis represents an important therapeutic target for ischemic heart disease. In the present study, SHED-CM attenuated myocyte apoptosis in response to I/R in a mouse model. SHED-CM also suppressed ischemia-induced cardiomyocyte apoptosis *in vitro*. Thus, the ability of SHED-CM to attenuate myocardial infarct size following I/R is dependent, at least in part, on its ability to reduce apoptosis in the heart.

HGF, originally purified as a potent mitogen for hepatocytes, has anti-apoptotic activities in various cells including cardiac myocytes^20^,^21^,^22^,^23^. Administration of recombinant HGF protein in rats reduced infarct area size and improved cardiac function by suppressing apoptosis in cardiac myocytes^24^. Neutralization of HGF results in increased infarct size and apoptosis in the heart. In the present study, SHED-CM attenuated ischemia-induced apoptosis more effectively than BMSC-CM or ADSC-CM in cardiac myocytes. Notably, SHED-CM contained a higher concentration of HGF than BMSC-CM and ADSC-CM, and depletion of HGF attenuated the inhibitory actions of SHED-CM on apoptosis *in vitro* and *in vivo*. Thus, SHED-CM protects the heart from ischemic injury through its ability to promote pro-survival pathways involving HGF signaling. We also confirmed that SHED-CM contained various cytokines in addition to HGF by cytokine antibody array analysis. Combined effects of these factors in SHED-CM could provide cardioprotective benefits against acute cardiac injury.

In conclusion, in the present study we show that dental pulp-derived stem cell conditioned medium protects the heart from injury in response to I/R through at least two mechanisms: improvements in myocardial cell viability and suppression of inflammatory cytokines in mice. Clearly, additional investigations using larger animal species are warranted, but our current method may provide a novel therapeutic strategy for acute myocardial infarction in the near future.

## Material and Methods

### SHEDs, BMSCs, and ADSCs culture and preparation of conditioned media

Human SHEDs were isolated as described previously^6^. Briefly, exfoliated deciduous teeth from 6- to 12-year-old individuals were extracted for clinical purposes and collected at Nagoya University School of Medicine. After separating the crown and root, the dental pulp was isolated and digested in a solution of collagenase type I (3 mg/ml) and dispase (4 mg/ml) for 1 h at 37 °C. Single-cell suspensions (1 × 10^4^ to 2 × 10^4^ cells/ml) were plated on culture dishes in Dulbecco’s Modified Eagle Medium (DMEM) supplemented with 10% fetal bovine serum (FBS), then incubated at 37 °C in 5% CO_2_. SHEDs expressed a set of MSC markers (i.e., CD90, CD73, and CD105) but not endothelial and hematopoietic markers (i.e., CD34, CD45, CD11b/c, or HLA-DR). MSCs originated from human bone marrow (BMSCs) and human adipose tissue (ADSCs) were obtained from Lonza Ltd. SHEDs, BMSCs, and ADSCs were used at passage 8–10. At 70–80% confluence, these cells were washed with phosphate-buffered saline (PBS), the culture media were replaced with serum-free DMEM. After 48-h incubation, the media were collected and centrifuged for 3 min at 440 × *g* and 4 °C to remove detached cells, and the supernatants were then collected and centrifuged for 3 min, at 1,740 × *g* and 4 °C. These supernatants were collected and used for assays as SHED-CM, BMSC-CM, and ADSC-CM.

### Mouse model of myocardial ischemia-reperfusion injury

Male C57BL/6 J mice were purchased from Japan SLC, Inc. (Shizuoka, Japan). At 8 to 12 weeks of age, mice were subjected to myocardial ischemia-reperfusion (I/R) as described previously^25^,^26^. Briefly, after anesthetization (pentobarbital sodium 50 mg/kg, intraperitoneally) and intubation, we performed left thoracotomy and visualized the left anterior descending artery (LAD) under a microscope. The LAD was ligated for 30 min with 8-0 nylon suture using a snare occluder, which was then loosened for reperfusion. Five minutes after following reperfusion, 500 μl of SHED-CM or DMEM (as control) was infused systemically via jugular vein.

We performed transthoracic echocardiography to evaluate cardiac function of mice at 7 days after I/R surgery. Left ventricular (LV) end diastolic diameter (LVEDD) and LV end systolic diameter (LVESD) were measured by M-mode images using an Acuson Sequioa C-256 machine with a 15-MHz probe, and LV fractional shortening was calculated as (LVEDD-LVESD)/LVEDD × 100 (%)^25^.

### Determination of area at risk and infarct size

At 24 h after reperfusion, the suture of the LAD artery was re-tied, and 1 ml of 1.0% Evans blue dye (Sigma-Aldrich Co. LLC) was injected into the mice systemically through the jugular vein to delineate the nonischemic tissue. Then, the hearts were excised, washed with PBS, cut into four transverse slices, and incubated in 1.5% 2,3,5-triphenyltetrazolium chloride solution (TTC, Sigma-Aldrich Co. LLC) for 15 min at room temperature to determine the infarct region. The heart sections were weighed and photographed under a microscope, and left ventricular area (LV), the area at risk (AAR), and infarct area (IA) were assessed by computerized planimetry using NIH ImageJ software. The infarct size was expressed as a percentage of the IA to the AAR and LV^25^,^26^.

At 24 h after myocardial ischemia-reperfusion, plasma was collected from the mice, and the circulating levels of cardiac troponin-I were determined as a marker of myocardial injury using a high-sensitivity mouse cardiac troponin-I ELISA kit (Life Diagnostics, Inc.) according to the manufacturer’s instructions^27^.

### Cultures of cardiac myocytes and treatment with conditioned media

Primary cultures of neonatal rat ventricular myocytes were prepared and incubated in DMEM supplemented with 10% FBS as described previously^25^,^26^. Culture media were replaced with SHED-CM, BMSC-CM, ADSC-CM, or fresh DMEM respectively. For the study of hypoxia and serum deprivation, cardiac myocytes were incubated under conditions of hypoxia for 24 h (<1% O_2_ and 5% CO_2_, 37 °C) and normoxia for 48 h (21% O_2_ and 5% CO_2_, 37 °C). The hypoxic condition was generated with a CO_2_ incubator (9000EX, Wakenbitech Co, Ltd.). Apoptotic myocytes and cell viability were examined after 24-h hypoxia and 48-h normoxia. In some experiments, after 12-h serum-free culture, media were replaced with SHED-CM, BMSC-CM, ADSC-CM, or DMEM (as control), and cardiac myocytes were subjected to lipopolysaccharide (LPS) stimulation (100 ng/ml) for 6 h to induce inflammation.

### Determination of mRNA expression of pro-inflammatory cytokines

Gene expression was quantified by real-time polymerase chain reaction (PCR). Total RNA was extracted from heart tissue using Isogen II (Nippon Gene Co., Ltd.), and from cardiac myocytes using RNeasy Micro Kit (Qiagen). Reverse transcription was carried out with M-MLV Reverse Transcriptase (Invitrogen). Real-time PCR was performed using the Thunderbird SYBR qPCR Mix (TOYOBO Co., Ltd.) driven by the StepOnePlus Real-Time PCR System (Applied Biosystems). We used the primers listed in Supplementary Table 1.

### Determination of apoptosis

TUNEL staining of frozen heart sections and cultured cardiac myocytes was performed using an In Situ Cell Death Detection Kit with TMR red (Roche Applied Science) as described previously^25^,^26^. Cryosections (5-μm thick) embedded in O.C.T. compound (Sakura Finetek Japan Co., Ltd) were fixed with 4% paraformaldehyde in PBS and permeabilized with 0.1% Triton X-100. Anti-sarcomeric α-actinin antibody was used for determination of myocytes followed by TUNEL and nuclear staining. DAPI (Sigma-Aldrich Co. LLC) was used for nuclear staining. Three randomly chosen microscopic fields from five different sections in each tissue block were examined for the presence of TUNEL-positive cells.

### Cell viability assay

Cell viability was assayed using the Cell Counting Kit-8 (Dojindo Molecular Technologies, Inc., Kumamoto, Japan) according to the manufacturer’s instructions. Briefly, cardiac myocytes were cultured with conditioned media or vehicle for 48 h, then water-soluble tetrazolium salt (WST-8) was added for 2 h. The culture medium was collected and measured at a wavelength of 450 nm absorbance using a microplate reader (Tecan Japan Co., Ltd.) to analyze conversion of WST-8 into formazan by mitochondrial activity in living cells. Percentage viability was defined as the relative absorbance of all experimental groups.

### Cytokine antibody array

Cytokine antibody array experiments were carried out using the RayBio G-Series Human Cytokine Antibody Array 4000 kit (Applied Arrays, Ray Biotech, Inc.) at Filgen, Inc. (Nagoya, Japan)^7^. Using laser scanning, cytokines in SHED-CM, BMSC-CM, ADSC-CM and serum-free DMEM were detected from 274 human cytokine array plates. All scans were carried out in duplicate, and data were calculated by normalizing to the ratio of the level in conditioned medium to that in serum-free DMEM.

### Measurement of HGF in conditioned media, and HGF depletion assay

HGF levels in conditioned media were quantified with the Quantikine ELISA Human HGF Immunoassay kit (R & D Systems, Inc.). To deplete the SHED-CM of HGF, antibodies pre-attached to Protein G Sepharose (GE Healthcare) were added to the SHED-CM, the mixture was incubated overnight at 4 °C, and the antibody beads were removed by centrifugation.

### Study approval

The animal studies were carried out in accordance with the NIH Guidelines for the Care and Use of Laboratory Animals and approved by the Institutional Animal Care and Use Committee of Nagoya University. Exfoliated deciduous teeth were collected at Nagoya University Hospital, under approved guidelines set by Nagoya University (H-73, 2003). Ethical approval was obtained from the ethics committee of Nagoya University (permission number 8-2). All participants provided written informed consent.

### Statistical analysis

Data are presented as mean ± SEM. Group differences were analyzed by Mann-Whitney U-test. A value of *p* < 0.05 was considered to denote the presence of a statistically significant difference.

## Additional Information

**How to cite this article**: Yamaguchi, S. *et al.* Dental pulp-derived stem cell conditioned medium reduces cardiac injury following ischemia-reperfusion. *Sci. Rep.* **5**, 16295; doi: 10.1038/srep16295 (2015).

## Supplementary Material

Supplementary Information
