# Supplementary material for: Dental pulp-derived stem cell conditioned medium reduces cardiac injury following ischemia-reperfusion
Source: Sci Rep. 2015 Nov 6;5:16295. doi: 10.1038/srep16295 (PMC4635346; doi:10.1038/srep16295)
Supplement: Supplementary Information [file srep16295-s1.pdf]

Supplementary Information

Dental pulp-derived stem cell conditioned medium reduces cardiac injury following ischemia-reperfusion

Satoshi Yamaguchi, DDS[1], Rei Shibata, MD, PhD[2], Noriyuki Yamamoto, DDS, PhD[1], Masaya Nishikawa, DDS, PhD[1], Hideharu Hibi DDS, PhD[1], Tohru Tanigawa, MD, PhD[3], Minoru Ueda, DDS, PhD[1], Toyoaki Murohara, MD, PhD[2] and Akihito Yamamoto, DDS, PhD[1]

[1]Department of Oral and Maxillofacial Surgery, Nagoya University Graduate School of Medicine, Nagoya, Japan
[2]Department of Cardiology, Nagoya University Graduate School of Medicine, Nagoya, Japan
[3]Department of Otolaryngology, Aichi Medical University, Nagakute, Japan

Figure 2c

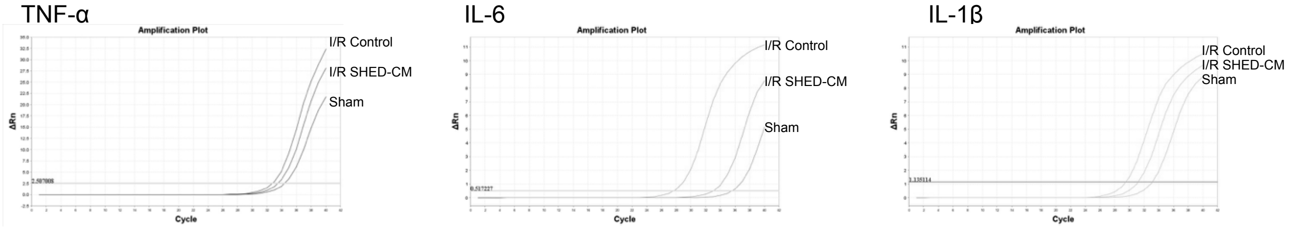

Figure 3d

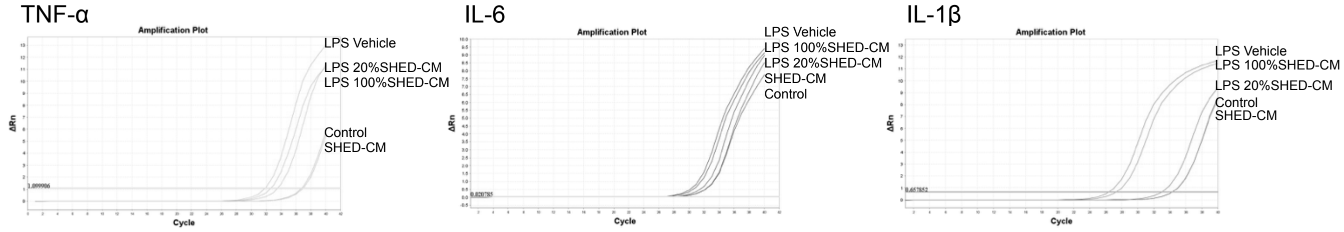

Figure 4c

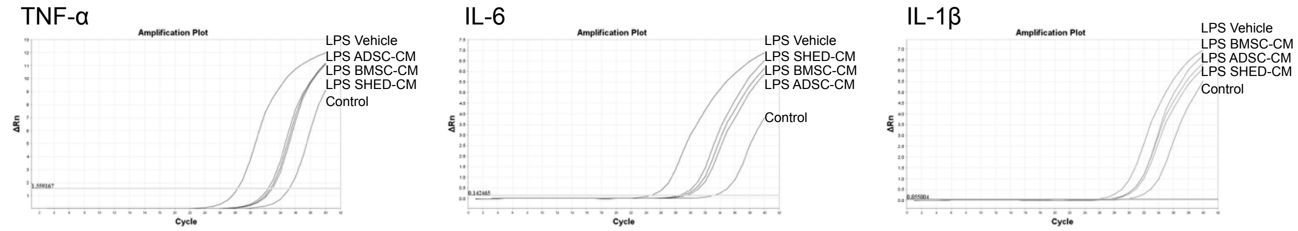

Figure 5e

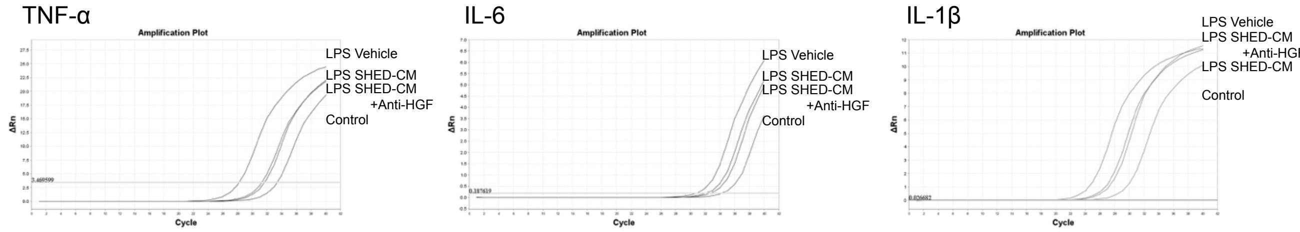

Supplemental Figure 1: Representative amplification curves in Figure 2C, Figure 3D, Figure 4C and Figure 5E.

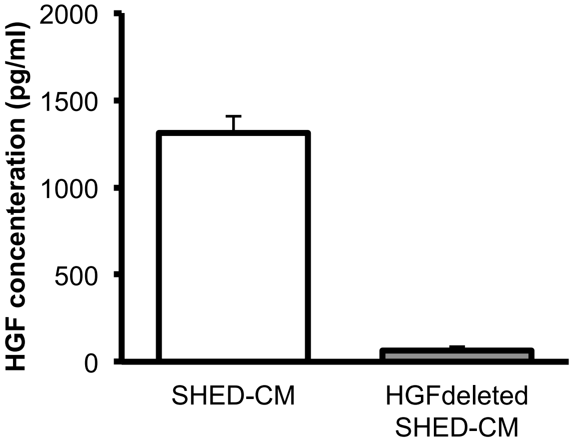

Supplemental Figure 2. Quantitative analysis of HGF concentrations in SHED-CM or HGF-depleted SHED-CM. HGF levels in conditioned media were quantified with the Quantikine ELISA Human HGF Immunoassay kit.

Supplemental Figure 2

Used primers for quantitative PCR

Mouse Primers		Sequence
TNF-α	forward	5’-CCCTTTACTCTGACCCCTTTATTGT-3’
	reverse	5’-TGTCCCAGCATCTTGTGTTTCT-3’
IL-6	forward	5’-CCAAGAACGATAGTCAATTCCAGA-3’
	reverse	5’-CATCAGTCCCAAGAAGGCAAC-3’
IL-1β	forward	5’-CAGGATGAGGACCCAAGCAC-3’
	reverse	5’-TCAGACAGCACGAGGCATTT-3’
GAPDH	forward	5’-AACTTTGGCATTGTGGAAGGT-3’
	reverse	5’-GGATGCAGGGATGATGTTCT-3’
**Rat Primers**		**Sequence**
TNF-α	forward	5’-CTCGAGTGACAAGCCCGTAG-3’
	reverse	5’-CCTTGAAGAGAACCTGGGAGTAG-3’
IL-6	forward	5’-TTGCCTTCTTGGGACTGATG-3’
	reverse	5’-ACTGGACTGTTGTGGGTGGT-3’
IL-1β	forward	5’-CAGGATGAGGACCCAAGCAC-3’
	reverse	5’-TCAGACAGCACGAGGCATTT-3’
GAPDH	forward	5’-AACTTTGGCATCGTGGAAGG-3’
	reverse	5’-CGGATACATTGGGGGTAGGA-3’

Supplemental Table 1
